# The Association between Diet Quality and Metabolic Syndrome among Older African American Women

**DOI:** 10.3390/nu16173040

**Published:** 2024-09-09

**Authors:** Alex Grant, Chiranjeev Dash, Lucile L. Adams-Campbell

**Affiliations:** 1 Graduate School of Art and Sciences, Epidemiology Program, Georgetown University, Washington, DC 22057, USA; adg111@georgetown.edu; 2 Georgetown Lombardi Comprehensive Cancer Center, Washington, DC 20003, USA; cd422@georgetown.edu

**Keywords:** diet, diet quality, metabolic syndrome, Black women

## Abstract

Diet is a modifiable lifestyle factor that could impact the development of Metabolic Syndrome (MetS) and its components. MetS prevalence is high and diet quality is suboptimal among older African American women. MetS has been associated with many individual food groups, however, emerging research suggests that analyzing overall diet quality provides insight into the synergistic effects of food groups on health outcomes. In the current cross-sectional study, we examined the relationship between diet quality and MetS, and investigated associations between diet quality and MetS components among older African American women. This study was based on 357 African American women between 45 and 65 years from the NHANES 2011–2018 datasets. This analysis utilized the NCEP ATP III (2001) criteria for women to diagnose MetS. MetS was dichotomized in addition to a MetS z-score being calculated for each participant using a sex- and race-specific equation. Participants’ diet quality was measured using the HEI-2015. Linear and logistic regressions were performed to assess the association between HEI-2015 diet quality and metabolic syndrome and its components. 65% of African American women aged 45–65 in the NHANES 2011–2018 had MetS. Study participants had an average HEI-2015 score of 55.4 out of 100. As HEI-2015 quartiles increased, the mean MetS z-score decreased (*p*-value: 0.0011). Age-adjusted models demonstrated statistically significant inverse relationships between HEI-2015 and waist circumference (β: −0.217; 95% CI: −0.372, −0.063), systolic blood pressure (β: −0.215; 95% CI: −0.359, −0.072), blood glucose (β: −0.344; 95% CI: −0.681, −0.0066), and triglycerides (β: −0.652; 95% CI: −1.05, −0.251). Significant associations could not be established between MetS and diet quality, assessed with the HEI-2015, among African American women aged 45–65 enrolled in NHANES 2011–2018. However, statistically significant relationships were observed between increased HEI-2015 scores and lowered risks of abdominal obesity, hyperglycemia, hypertriglyceridemia, and systolic hypertension. The findings of this study affirm the necessity of public health strategies to improve diet quality among African-American women which could help to reduce their risks of chronic diseases.

## 1. Introduction

Diet is a modifiable risk factor that plays a role in developing MetS components and CVDs such as type 2 diabetes and obesity [1,2]. The US Departments of HHS and Agriculture have collaborated to create the Dietary Guidelines for Americans (DGA) which aims to improve the diet of Americans through a set of recommendations on food/nutrient consumption levels. The DGA recommends that daily, Americans should consume 2 ½ cups equivalent of vegetables, 2 cups equivalent of fruits, 6-ounce equivalents of grains, 3 cups equivalent of grains, 5 ½-ounce equivalents of protein foods, and 27 g of oils. Additionally, Americans are encouraged to limit sodium consumption to less than 2300 mg per day, and foods high in saturated fats and added sugars should account for less than 10 percent of total calories per day [3]. However, most Americans do not adhere to these recommendations [3]. The typical American diet consists of low fruit and vegetable intake and is excess in sugars, fat, and sodium [4,5]. Racial and ethnic minority populations have even lower adherence to these guidelines. According to the Centers for Disease Control and Prevention (CDC), in 2019, only 6.9% of non-Hispanic Black adults met the guidelines for vegetable intake [6]. 12.5% of Hispanic adults met the recommended fruit intake level and this represents the highest intake levels across racial and ethnic groups [6]. Expectedly, failure to adhere to recommended intake levels for certain food groups has been associated with increased odds of MetS and its components [7,8,9]. Lepping et al., previously reported that African American women with poor adherence to fiber intake guidelines were 4.24 times more likely to have MetS compared to those who followed the intake guidelines.

Although MetS has been associated with many other individual food groups, emerging research suggests that analyzing overall diet quality provides insight into the synergistic effects of food groups on health outcomes [10]. Diet quality can be measured using several indices based either on dietary guidelines (e.g., the Healthy Eating Index) or dietary patterns associated with the risk of chronic diseases (e.g., the Mediterranean Diet Score, Healthful Plant-Based Diet Index, and the Dietary Approaches to Stop Hypertension) [11]. Some diet quality indices are also country-specific. The Healthy Eating Index (HEI) is a diet quality index designed to evaluate how well the US population adheres to the recommendations set by the DGA [12]. The HEI uses a scoring system which ranges between 0 and 100. A higher diet quality score indicates greater compliance with the DGA recommendations and in turn, a better diet quality. The 2015 and 2020 versions of the HEI have 13 dietary components which are divided into two categories, adequacy components which are to be consumed at higher levels, and moderation components which are consumed at lower levels [13]. The American diet quality is considered suboptimal as indicated by an HEI-2020 score of 58 out of 100 [12]. When race is considered, non-Hispanic Black adults are 16% more likely to have poor diet quality when compared to non-Hispanic White adults [14,15].

A limited number of studies have evaluated the relationship between MetS and diet quality [3,16]. The existing literature that examines this health issue is primarily among non-American populations and uses country-specific dietary recommendations. To our knowledge, there is currently no study that describes the diet quality of African American women with MetS nor examines the relationship between MetS components and diet quality using HEI-2015 among this group.

Previous reports have established that among African American women MetS prevalence is high [9,17]. Additionally, other studies have reported that the diet quality of African American women is suboptimal. Furthermore, it has been reported that the odds of being diagnosed with MetS increase by 73% for every 10-year increase in age [18,19]. Resulting from a paucity of research evaluating this public health issue, in a cross-sectional study we propose to: (1) examine the relationship between diet quality and MetS and; (2) investigate associations between diet quality and MetS components among older African American women. We hypothesize that an inverse relationship exists between MetS and diet quality among older African American women. We further hypothesize that there are significant associations between all MetS components and diet quality among older African American women.

## 2. Materials and Methods

### 2.1. Study Population

The cross-sectional study was based on 357 African American women between 45 and 65 years from the NHANES 2011–2018 datasets. NHANES is a program of studies that uses a stratified, multistage, and probability-cluster design to assess the health and nutritional statuses of Americans over 2 years old [20]. The survey examines roughly 5000 people each year which is nationally representative of the US population. The survey also uses its findings to assess the prevalence of major diseases and their risk factors and assesses the association between nutritional status and health promotion. NHANES received ethical approval from the National Center for Health Statistics Research Ethics Review Board (Protocol# 2011-17).

**Figure 1:**
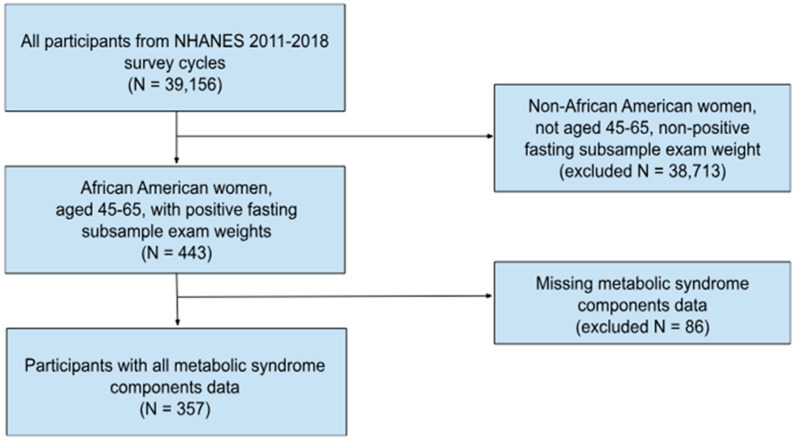
Consort diagram of African American women included from NHANES 2011–2018.

NHANES 2011–2018 datasets were merged and included 39,156 participants’ demographic, physical examination, laboratory tests, and nutrient intake information. Numerous participants from the initial search were missing some MetS components required to diagnose the condition. NHANES recommends using the subsample weight for the smallest subpopulation to account for the high number of missing data [21]. In this study, the fasting glucose had the smallest subpopulation, hence only participants with this subsample weight, hereon called a positive fasting subsample weight, were included. After selecting African American women aged 45–65 with a positive fasting subsample weight and not missing any MetS components, our study included 357 participants as shown in the consort diagram (Figure 1). No participants were lost after meeting these inclusion criteria.

### 2.2. Metabolic Syndrome Components Measurements, Diagnosis, and z-Score

This analysis utilized the National Cholesterol Education Program’s Adult Treatment Panel III (NCEP ATP III) (2001) [22] criteria for women to diagnose MetS: waist circumference ≥ 88 cm, blood pressure ≥ 130/85 mmHg, blood glucose ≥ 100 mg/dL, triglycerides ≥ 150 mg/dL, and HDL-cholesterol < 50 mg/dL. According to the NCEP ATP III, women meeting ≥3 of these criteria are diagnosed with MetS [22]. Specific methods regarding the measurement of these variables have been previously reported in the NHANES 2011–2018 laboratory and physical examination documentation files [23]. In this study, each MetS component was analyzed continuously and categorically (high versus low) using the criteria outlined in Table 1. Using the NCEP ATP III criteria, MetS was dichotomized into ‘MetS present’, consisting of participants having ≥3 MetS components, and ‘MetS absent’, which included participants with <3 MetS components. Additionally, a continuous MetS z-score was calculated for each participant using the sex- and race-specific equation below. The MetS z-score signifies a person’s risk or severity of MetS. A higher MetS z-score indicates a higher risk or severity of MetS, while the opposite is true for a lower MetS z-score.

### 2.3. Dietary Intake and Diet Quality Assessment

NHANES 2011–2018 dietary intake information was collected in collaboration with the US Department of Agriculture using the Automated Multiple-Pass Method [25]. NHANES collected two sets of dietary intake data from participants, one in person and the other over the phone. Participants’ diet quality was measured using the Healthy Eating Index (HEI) 2015 [12] which indicates the degree to which their food intake aligns with the recommendations of the DGA [13]. This overall score ranges between 0 and 100, with a higher score indicating better diet quality and greater adherence to the DGA. Similarly, a higher food/nutrient component score indicates greater adherence to the dietary guidelines for those components. A detailed explanation of the HEI-2015 scoring methodology is provided by the National Cancer Institute’s Division of Cancer Control and Population Sciences [26]. The simple scoring method-per person was used to calculate the HEI-2015 for NHANES 2011–2018 participants as there were two days of nutrient intake (DR1TOT and DR2TOT) available. The sample SAS codes provided by the National Cancer Institute were used to calculate HEI-2015 scores [26]. The resulting HEI-2015 scores were examined continuously and in quartiles for this analysis.

### 2.4. Covariates

Self-reported demographic and lifestyle factors such as age, educational level, income, and smoking status were the covariates included in this analysis. All covariates, except smoking status, were re-categorized. A relatively high number of income data was missing; therefore, income was not included in the multivariable logistic regression models to avoid potential bias.

### 2.5. Statistical Analysis

Frequency tables were generated to provide descriptive statistics of participants. An univariate analysis was conducted on all continuous MetS components and the MetS z-score to understand the distribution of variables. For variables that were normally distributed, one-way ANOVA tests were used to determine whether statistically significant differences existed between the outcome variables (metabolic syndrome z-score and components) and the HEI-2015 quartiles. Kruskal-Wallis tests were used if the outcome variables were non-parametric. Chi-square tests were employed to determine if statistically significant differences existed between categorical outcome variables and HEI-2015 quartiles. Interaction assessments were conducted between HEI-2015 and the covariates. If the interactions were statistically significant, they were included in logistic regression models. Logistic regressions were performed to assess the association between HEI-2015 diet quality and metabolic syndrome and its components. Linear regressions were performed to assess the relationship between metabolic syndrome z-score and HEI-2015 as a continuous variable. Variables that had non-normally distributed residuals were log-transformed before conducting linear regression analyses. Variables with negative or zero values were translated and then log-transformed for analysis. Forest plots were created to visualize associations using the MedCalc software (Version 22.021). All statistical analyses were conducted using SAS software (Version 9.4; SAS Institute, Cary, NC, USA).

## 3. Results

The average age of this study population was 55.2 years. 65% of African American women aged 45–65 in the NHANES 2011–2018 had MetS. Two-thirds of African American women diagnosed with MetS had a high school diploma/some college education and 44% had an income less than $35,000. Table 1 further describes the baseline characteristics of the study population.

Study participants had an average HEI-2015 score of 55.4 (Table 2). The minimum HEI-2015 score was 18.2 while the maximum was 87.3. Participants had high adherence to the dietary guidelines for total protein foods component only, with 92.8% meeting those recommendations. However, less than 36% of women adhered to the dietary guidelines for dairy and whole grains. Sodium intake was also poor with only 40% meeting the standard. The other food/nutrient components had 50–70% of women adhering to intake recommendations.

Only 23% of participants with MetS had an HEI-2015 score in the highest quartile, Quartile 4 (HEI-2015 range: 63.89–87.29). Additionally, 54% of women without MetS had HEI-2015 scores in the highest quartiles, Quartiles 3 and 4 (Table 3). As HEI-2015 quartiles increased, the mean MetS z-score decreased. The difference in the mean MetS z-score by quartiles was statistically significant (*p*-value: 0.0011).

There were no statistically significant two-way interaction assessments found between HEI-2015 and covariates. Therefore, the two-way interactions were omitted and the covariates were placed in the models to control for possible confounding. Logistic regressions were conducted to determine the effect size of associations between MetS and HEI-2015.

Age-adjusted models demonstrated statistically significant inverse relationships between HEI-2015 and waist circumference (β: −0.217; 95% CI: −0.372, −0.063), systolic blood pressure (β: −0.215; 95% CI: −0.359, −0.072), blood glucose (β: −0.344; 95% CI: −0.681, −0.0066), and triglycerides (β: −0.652; 95% CI: −1.05, −0.251) (Table 4). The *p*-values of these linear regressions were <0.05.

## 4. Discussion

African American women 45–65 years old a part of NHANES 2011–2018 had a suboptimal diet quality as reflected by an average HEI-2015 score of 55.4 out of 100. Significant associations could not be established between MetS and diet quality, assessed with the HEI-2015, among African American women aged 45–65 enrolled in NHANES 2011–2018. However, statistically significant relationships were observed between increased HEI-2015 scores and lowered risks of abdominal obesity, hyperglycemia, hypertriglyceridemia, and systolic hypertension.

To our knowledge, this is the first cross-sectional study to investigate the association between overall diet quality and MetS among African-American women aged 45 and 65 years. Previous studies among different populations have presented conflicting information on MetS’s relationship with diet quality [27,28,29,30]. However, similar to our findings, most studies have deduced that diet quality impacts certain MetS components. An observational prospective study concluded that having higher HEI-2010 diet quality scores was associated with lowered waist circumference, blood glucose levels, triglyceride, and higher HDL-cholesterol among Mexican-descent women [30]. Comparably, Yosaee and colleagues found that among a population of Iranian women, with increased diet quality there were significant reductions in triglycerides, blood pressure, and body mass index [31].

Our study findings highlight the possible health benefits of improving diet quality, particularly for Black women. We found that every unit increase in diet quality was associated with statistically significant reductions in waist circumference, systolic blood pressure, blood glucose, and triglycerides. This indicates a possible protective effect of diet quality on MetS components that disproportionately affect Black women; namely abdominal obesity, hypertension, and hyperglycemia. Moreover, limiting the development of these MetS components through improved diet quality could also lower the risks of CVDs and cancers like those associated with obesity.

The findings of this study also affirm the necessity of public health strategies to improve areas of Black women’s diet quality which could help to reduce their risks of chronic diseases and cancers. Our study showed that Black women’s overall diet quality is suboptimal which could be due to less than 70% of women adhering to the DGA intake recommendations for 12 out of 13 components. It would be beneficial to formulate targeted interventions that assist Black women in increasing their intake of dietary components like fruits, vegetables, and whole grains which previous reports have noted can reduce the likelihood of coronary diseases and cancers [32,33,34,35].

There were several strengths of this study. First, we used data from a large, nationally representative database, NHANES. Data from four NHANES cycles were used to increase our study’s sample size. Using the most recent available version of the HEI to assess diet quality is a major strength of this study. However, our study had several limitations. This study was cross-sectional by design, therefore changes in diet quality or metabolic syndrome could not be analyzed. Also, this study did not analyze physical activity levels and income levels as potential confounding variables. Furthermore, we did not assess the number of participants undergoing hormone replacement therapy which could interfere with our results.

In future studies, it would be beneficial to analyze how changes in African American women’s diet quality over time impact metabolic syndrome components and z-scores. Studies could also investigate the relationship between having varying numbers of metabolic syndrome components, not only 3 or more, and diet quality. Additionally, further analyses are needed to assess the relationship between HEI-2015 components and metabolic syndrome components among African American women.

## 5. Conclusions

The present study offers a novel understanding of the relationship between MetS and diet quality among older African American women. We found no significant associations between MetS and diet quality. However, statistically significant relationships exist between increased diet quality, measured with HEI-2015, and lowered risks of abdominal obesity, hyperglycemia, hypertriglyceridemia, and systolic hypertension. Further research analyzing how changes in African American women’s diet quality over time impact metabolic syndrome components and z-scores is necessary.

## Figures and Tables

**Table 1:** Baseline characteristics and MetS prevalence of African American women in NHANES 2011–2018.

Characteristics	n (%)	MetS Prevalence	
		MetS Present n (%) 233 (65.27)	MetS Absent n (%) 124 (34.73)
Age, mean (SD)	55.2 (5.96)		
Age (categorical), n(%)			
45–49	87 (24.37)	51 (21.88)	36 (29.03)
50–54	93 (26.05)	66 (28.33)	27 (21.77)
55–59	90 (25.21)	61 (26.18)	29 (23.39)
60–65	87 (24.37)	55 (23.61)	32 (25.81)
Education level, n(%)			
≤High school	40 (11.20)	32 (13.73)	8 (6.45)
High school/some college	233 (65.27)	153 (65.67)	80 (64.52)
≥College	84 (23.53)	48 (20.60)	36 (29.03)
Income level, n(%)			
≤$34,999	145 (40.62)	103 (44.21)	42 (33.87)
$35,000–$74,999	105 (29.41)	67 (28.76)	38 (30.65)
≥$75,000	73 (20.45)	39 (16.74)	34 (27.42)
Missing	34 (9.52)	24 (10.29)	10 (8.06)
Smoking (in lifetime), n(%)			
≥100 cigs.	142 (39.78)	98 (42.06)	44 (35.48)
Did not smoke 100 cigs.	215 (60.22)	135 (57.94)	80 (64.52)
Weighted frequencies using constructed 8-year fasting subsample weights			

MetS z-score = [(waist circumference − 88)/13.72] + [(fasting blood glucose − 100)/13.04] + [(triglycerides − 150)/61.17] + [(mean arterial pressure − 100)/11.26] + [(50 − HDL-cholesterol)/18.22] [9,24]; Mean arterial pressure = [{(systolic blood pressure) + (diastolic blood pressure ∗ 2)}/3] [9,24].

**Table 2:** HEI-2015 component and total scores for African American women in NHANES 2011–2018.

Food/Nutrient Component	N	Min.	Max.	Mean *	SE	Percent Score
Total Vegetables		0	5	3.41	0.07	68.2
Greens & Beans		0	5	2.31	0.11	46.2
Total Fruit		0	5	2.62	0.10	52.4
Whole Fruit		0	5	2.64	0.11	52.8
Whole Grains		0	10	2.88	0.15	28.8
Dairy		0	10	3.56	0.13	35.6
Total Protein Foods		0	5	4.64	0.04	92.8
Seafood & Plant Proteins		0	5	3.04	0.10	60.8
Fatty Acid Ratio		0	10	6.49	0.16	64.9
Sodium		0	10	4.07	0.15	40.7
Refined Grains		0	10	6.97	0.15	69.7
Saturated Fats		0	10	6.38	0.15	63.8
Added Sugars		0	10	6.36	0.16	63.6
**Total HEI-2015 Score**	**357**	**18.2**	**87.3**	**55.4**	**0.63**	**55.4**

* Weighted and calculated using the simple scoring method; The bold was added to highlight these findings as the major results.

**Table 3:** Association between MetS, MetS z-score, and HEI-2015 among African American women in NHANES 2011–2018.

HEI-2015 Quartiles	Metabolic Syndrome				
	MetS Present *n* (%)	MetS Absent *n* (%)	*p*-Value	MetS z-Score, Mean	*p*-Value
Quartile 1 (18.19–45.66)	61 (26.18)	29 (23.39)	0.6691	0.856	0.0011 *
Quartile 2 (45.67–54.86)	61 (26.18)	28 (22.58)		0.737	
Quartile 3 (54.87–63.87)	57 (24.46)	32 (25.81)		−0.512	
Quartile 4 (63.89–87.29)	54 (23.18)	35 (28.23)		−1.05	

Chi-square tests and one-way ANOVA tests were used appropriately to determine the *p*-value between groups. *p*-value < 0.05 indicates statistical significance.* Kruskal-Wallis test used to determine the *p*-value between groups.

**Table 4:** Linear regression models of HEI-2015 score and metabolic syndrome components among African American women in NHANES 2011–2018.

MetS Components	Unadjusted β (95% CI)	*p*	Age-Adjusted β (95% CI)	*p*	Multivariable ^1^ β (95% CI)	*p*
Waist circumference	**−0.216 (−0.371, −0.060) ^a^**	**0.0075 ^a^**	**−0.217 (−0.372, −0.063) ^a^**	**0.0067 ^a^**	**−0.212 (−0.377, −0.046) ^a^**	**0.013 ^a^**
Systolic blood pressure	**−0.226 (−0.381, −0.071) ^a^**	**0.0051 ^a^**	**−0.215 (−0.359, −0.072) ^a^**	**0.0039 ^a^**	**−0.205 (−0.348, −0.061) ^a^**	**0.006 ^a^**
Diastolic blood pressure	−0.076 (−0.177, 0.025)	0.1361	−0.0783 (−0.178, 0.022)	0.1218	−0.0758 (−0.175, 0.023)	0.1301
Blood glucose	**−0.353 (−0.694, −0.012) ^a^**	**0.0430 ^a^**	**−0.344 (−0.681, −0.0066) ^a^**	**0.0458 ^a^**	−0.312 (−0.644, 0.0199)	0.065
Triglycerides	**−0.663 (−1.06, −0.26) ^a^**	**0.0016 ^a^**	**−0.652 (−1.05, −0.251) ^a^**	**0.0019 ^a^**	**−0.543 (−0.939, −0.148) ^a^**	**0.008 ^a^**
HDL-cholesterol	0.0013 (−0.0007, 0.0033)	0.1956	0.0013 (−0.0007, 0.0033)	0.1954	0.0012 (−0.0008, 0.0032)	0.2407

^1^: Adjusted for age, education level, smoking status. *p*: *p*-value. ^a^: *p* < 0.05 signifies statistical significance. The bold was added to highlight these findings as the major results.

## Data Availability

The data presented in this study are available in the National Health and Nutrition Examination Survey (NHANES) repository at https://wwwn.cdc.gov/nchs/nhanes/ (accessed on 13 August 2024). These data were derived from the following resources available in the public domain: NHANES 2011–2012 available at “https://wwwn.cdc.gov/nchs/nhanes/continuousnhanes/default.aspx?BeginYear=2011” (accessed on 13 August 2024). NHANES 2013–2014 available at “https://wwwn.cdc.gov/nchs/nhanes/continuousnhanes/default.aspx?BeginYear=2013” (accessed on 13 August 2024). NHANES 2015–2016 available at “https://wwwn.cdc.gov/nchs/nhanes/continuousnhanes/default.aspx?BeginYear=2015” (accessed on 13 August 2024). NHANES 2017–2018 available at “https://wwwn.cdc.gov/nchs/nhanes/continuousnhanes/default.aspx?BeginYear=2017” (accessed on 13 August 2024).

## References

[1] Lacroix S., Cantin J., Nigam A. (2017). Contemporary issues regarding nutrition in cardiovascular rehabilitation. Ann. Phys. Rehabil. Med..

[2] Poor Nutrition|CDC (n.d.). Centers for Disease Control and Prevention. https://www.cdc.gov/chronic-disease/index.html.

[3] U.S. Department of Agriculture, U.S. Department of Health and Human Services Dietary Guidelines for Americans, 2020–2025, 9th ed.; December 2020. https://health.gov/our-work/nutrition-physical-activity/dietary-guidelines/current-dietary-guidelines.

[4] Rakhra V., Galappaththy S.L., Bulchandani S., Cabandugama P.K. (2020). Obesity and the Western Diet: How We Got Here. Mo. Med..

[5] Quader Z.S., Zhao L., Gillespie C., Cogswell M.E., Terry A.L., Moshfegh A., Rhodes D. (2017). Sodium Intake among Persons Aged ≥2 Years—United States, 2013–2014. Mmwr. Morb. Mortal. Wkly. Rep..

[6] Lee S.H., Moore L.V., Park S., Harris D.M., Blanck H.M. (2022). Adults Meeting Fruit and Vegetable Intake Recommendations—United States, 2019. Mmwr. Morb. Mortal. Wkly. Rep..

[7] Saklayen M.G. (2018). The Global Epidemic of the Metabolic Syndrome. Curr. Hypertens. Rep..

[8] Seo E.H., Kim H., Kwon O. (2019). Association between Total Sugar Intake and Metabolic Syndrome in Middle-Aged Korean Men and Women. Nutrients.

[9] Lepping K., Adams-Campbell L.L., Hicks J., Mills M., Dash C. (2022). Dietary fiber intake and metabolic syndrome in postmenopausal African American women with obesity. PLoS ONE.

[10] Hu F.B. (2002). Dietary pattern analysis: A new direction in nutritional epidemiology. Curr. Opin. Lipidol..

[11] Petersen K.S., Kris-Etherton P.M. (2021). Diet Quality Assessment and the Relationship between Diet Quality and Cardiovascular Disease Risk. Nutrients.

[12] Healthy Eating Index (HEI)|Food and Nutrition Service USDA Food and Nutrition Service. https://www.fns.usda.gov/cnpp/healthy-eating-index-hei.

[13] Reedy J., Lerman J.L., Krebs-Smith S.M., Kirkpatrick S.I., Pannucci T.E., Wilson M.M., Subar A.F., Kahle L.L., Tooze J.A. (2018). Evaluation of the Healthy Eating Index-2015. J. Acad. Nutr. Diet..

[14] Kibe L.W., Bazargan M., Bosah A., Schrode K.M., Kuo Y., Andikrah E., Shaheen M. (2023). Diet Quality of Older African Americans: Impact of Knowledge and Perceived Threat of COVID-19. Int. J. Environ. Res. Public Health.

[15] McCullough M.L., Chantaprasopsuk S., Islami F., Rees-Punia E., Um C.Y., Wang Y., Leach C.R., Sullivan K.R., Patel A.V. (2022). Association of Socioeconomic and Geographic Factors with Diet Quality in US Adults. JAMA Netw. Open.

[16] Konikowska K., Bombała W., Szuba A., Różańska D., Regulska-Ilow B. (2022). Metabolic Syndrome Is Associated with Low Diet Quality Assessed by the Healthy Eating Index-2015 (HEI-2015) and Low Concentrations of High-Density Lipoprotein Cholesterol. Biomedicines.

[17] Gaillard T.R. (2018). The Metabolic Syndrome and Its Components in African-American Women: Emerging Trends and Implications. Front. Endocrinol..

[18] Bentley-Lewis R., Koruda K., Seely E.W. (2007). The metabolic syndrome in women. Nat. Clin. Pract. Endocrinol. Metab..

[19] Hirode G., Wong R.J. (2020). Trends in the Prevalence of Metabolic Syndrome in the United States, 2011–2016. JAMA.

[20] Centers for Disease Control and Prevention (CDC). National Center for Health Statistics (NCHS) National Health and Nutrition Examination Survey Data. Hyattsville, MD: U.S. Department of Health and Human Services, Centers for Disease Control and Prevention. https://www.cdc.gov/nchs/nhanes/about_nhanes.htm.

[21] NHANES Tutorials—Weighting Module. (n.d.). CDC. https://wwwn.cdc.gov/nchs/nhanes/tutorials/weighting.aspx.

[22] Expert Panel on Detection, Evaluation, and Treatment of High Blood Cholesterol in Adults (2001). Executive Summary of the Third Report of the National Cholesterol Education Program (NCEP) Expert Panel on Detection, Evaluation, and Treatment of High Blood Cholesterol in Adults (Adult Treatment Panel III). JAMA.

[23] Centers for Disease Control and Prevention (CDC). National Center for Health Statistics (NCHS) National Health and Nutrition Examination Survey Questionnaire, Examination Protocol, and Laboratory Protocol). Hyattsville, MD: U.S. Department of Health and Human Services, Centers for Disease Control and Prevention. https://wwwn.cdc.gov/nchs/nhanes/.

[24] Gurka M.J., Ice C.L., Sun S.S., DeBoer M.D. (2012). A confirmatory factor analysis of the metabolic syndrome in adolescents: An examination of sex and racial/ethnic differences. Cardiovasc. Diabetol..

[25] USDA AMPM—USDA Automated Multiple-Pass Method. USDA ARS. https://www.ars.usda.gov/northeast-area/beltsville-md-bhnrc/beltsville-human-nutrition-research-center/food-surveys-research-group/docs/ampm-usda-automated-multiple-pass-method/.

[26] HEI Scoring Algorithm|EGRP/DCCPS/NCI/NIH. (13 May 2022). Epidemiology & Genomics Research Program. https://epi.grants.cancer.gov/hei/hei-scoring-method.html.

[27] Konikowska K., Bombała W., Szuba A., Różańska D., Regulska-Ilow B. (2023). A High-Quality Diet, as Measured by the DASH Score, Is Associated with a Lower Risk of Metabolic Syndrome and Visceral Obesity. Biomedicines.

[28] Saraf-Bank S., Haghighatdoost F., Esmaillzadeh A., Larijani B., Azadbakht L. (2017). Adherence to Healthy Eating Index-2010 is inversely associated with metabolic syndrome and its features among Iranian adult women. Eur. J. Clin. Nutr..

[29] Kahrizsangi M.A., Jafari F., Najam W., Safarpour A.R., Fattahi M.R., Nouri M., Ghalandari H., Askarpour M., Shirazi M.H., Amini M.R. (2023). Adherence to a healthy diet and odds of metabolic syndrome: A cross-sectional study. Clin. Nutr. ESPEN.

[30] Santiago-Torres M., Shi Z., Tinker L.F., Lampe J.W., Allison M.A., Barrington W., Crane T.E., Garcia D.O., Hayden K.M., Isasi C.R. (2020). Diet quality indices and risk of metabolic syndrome among postmenopausal women of Mexican ethnic descent in the Women's Health Initiative Observational Study. Nutr. Healthy Aging.

[31] Yosaee S., Erfani M.R., Bazrafshan M.R., Entezami N., Alinavaz M., Akbari M., Soltani S., Djafarian K. (2017). Correlation between Diet Quality and Metabolic Syndrome. J. Nutr. Food Secur..

[32] Aune D., Keum N., Giovannucci E., Fadnes L.T., Boffetta P., Greenwood D.C., Tonstad S., Vatten L.J., Riboli E., Norat T. (2016). Whole grain consumption and risk of cardiovascular disease, cancer, and all cause and cause specific mortality: Systematic review and dose-response meta-analysis of prospective studies. BMJ.

[33] Harris K.A., Kris-Etherton P.M. (2010). Effects of whole grains on coronary heart disease risk. Curr. Atheroscler. Rep..

[34] Aune D., Giovannucci E., Boffetta P., Fadnes L.T., Keum N., Norat T., Greenwood D.C., Riboli E., Vatten L.J., Tonstad S. (2017). Fruit and vegetable intake and the risk of cardiovascular disease, total cancer and all-cause mortality-a systematic review and dose-response meta-analysis of prospective studies. Int. J. Epidemiol..

[35] Zurbau A., Au-Yeung F., Blanco Mejia S., Khan T.A., Vuksan V., Jovanovski E., Leiter L.A., Kendall C.W.C., Jenkins D.J.A., Sievenpiper J.L. (2020). Relation of Different Fruit and Vegetable Sources with Incident Cardiovascular Outcomes: A Systematic Review and Meta-Analysis of Prospective Cohort Studies. J. Am. Heart Assoc..

